# Development of a novel walkability index for London, United Kingdom: cross-sectional application to the Whitehall II Study

**DOI:** 10.1186/s12889-016-3012-2

**Published:** 2016-05-18

**Authors:** Jemima C. Stockton, Oliver Duke-Williams, Emmanuel Stamatakis, Jennifer S. Mindell, Eric J. Brunner, Nicola J. Shelton

**Affiliations:** Research Department of Epidemiology and Public Health, UCL (University College London), 1-19 Torrington Place, London, WC1E 6BT UK; Department of Information Studies, Foster Court, UCL, London, WC1E 6BT UK; Charles Perkins Center, Prevention Research Collaboration, School of Public Health, Sydney Medical School, University of Sydney, Sydney, Australia; Faculty of Health Sciences, University of Sydney, Sydney, Australia

**Keywords:** Geographic information systems, London, Neighbourhood, Walkability index, Walking

## Abstract

**Background:**

Physical activity is essential for health; walking is the easiest way to incorporate activity into everyday life. Previous studies report positive associations between neighbourhood walkability and walking but most focused on cities in North America and Australasia. Urban form with respect to street connectivity, residential density and land use mix—common components of walkability indices—differs in European cities. The objective of this study was to develop a walkability index for London and test the index using walking data from the Whitehall II Study.

**Methods:**

A neighbourhood walkability index for London was constructed, comprising factors associated with walking behaviours: residential dwelling density, street connectivity and land use mix. Three models were produced that differed in the land uses included. Neighbourhoods were operationalised at three levels of administrative geography: (i) 21,140 output areas, (ii) 633 wards and (iii) 33 local authorities. A neighbourhood walkability score was assigned to each London-dwelling Whitehall II Study participant (2003–04, *N* = 3020, mean ± SD age = 61.0 years ± 6.0) based on residential postcode. The effect of changing the model specification and the units of enumeration on spatial variation in walkability was examined.

**Results:**

There was a radial decay in walkability from the centre to the periphery of London. There was high inter-model correlation in walkability scores for any given neighbourhood operationalisation (0.92–0.98), and moderate-high correlation between neighbourhood operationalisations for any given model (0.39–0.70). After adjustment for individual level factors and area deprivation, individuals in the most walkable neighbourhoods operationalised as wards were more likely to walk >6 h/week (OR = 1.4; 95 % CI: 1.1–1.9) than those in the least walkable.

**Conclusions:**

Walkability was associated with walking time in adults. This walkability index could help urban planners identify and design neighbourhoods in London with characteristics more supportive of walking, thereby promoting public health.

## Background

### Physical environment and walking

Walking is a form of transport, with physical activity a healthy “side-effect”. An understanding of the physical environmental barriers and facilitators of this personal mobility is thus a prerequisite of creating neighbourhoods that improve public health. The relationships between urban form and physical activity have been examined in many cities of high income countries but most studies have focussed on cities in North America and Australia [1]. The urban form of London in the United Kingdom is likely to differ in many ways from these cities: London is significantly older and its growth has been constrained by a greenbelt, a land use policy to restrict urban growth. Public health concerns and industrialisation during the nineteenth and twentieth centuries in London led to dispersal of the population and a shift from a pedestrian-oriented transport network to one prioritizing motorised vehicles. Whilst this shift helped eradicate overcrowding-associated endemic infectious diseases and to transport people and goods faster, London’s rapid spatial evolution may have inadvertently driven the emergence of the non-infectious public health crises we see today. High blood pressure, obesity and overweight, and physical inactivity are major contributors to disability-adjusted life-years (DALYs) in England [2]. The enablement of excessive mobility, which permits travel over greater distances than on foot or by bicycle, probably reduced physical activity in individuals’ daily routines and increased obesity [3]: for example, countries with the highest levels of active transportation have the lowest obesity rates [4].

### Measures of walkability

Walking is associated with physical environmental attributes such as greater diversity in land use (land use mix) [5–7], greater street connectivity [8–10], and higher residential density [11, 12]. Greater land use mix is posited to enable better access to services and employment, and to induce shorter within-neighbourhood travel by foot when a range of destinations is located near residences [13]. In areas where different destinations such as restaurants and workplaces are co-located, walking is also likely to be more time-efficient than using public or private motorised transport to access them. Street connectivity relates to the feasibility of walking from one point to another: the more connected the streets, the more direct the route through the network and the greater the walkability [14]. Higher residential density is proposed to create a more walkable environment by providing a critical mass of walkers seen by other people who are, in turn, encouraged by safety in numbers to walk as well [15]. Also, traffic congestion associated with higher residential density may promote active above non-active travel [12].

Attributes of the physical environment that are associated with walking often co-exist; historically many researchers quantified a single attribute as a proxy for walkability, defined as the extent to which a place supports walking and cycling as physically active forms of transport and recreation. However, a consensus is growing that physical environmental attributes should not be measured in isolation because they do not always reflect one another, and may be insufficient individually to promote physical activity [16]. For example, greater street connectivity may be relevant only if people have a range of places with complementary uses to visit—greater land use mix [17].

Walkability indices are designed to reflect these various elements by capturing the multiple attributes of a place for which there is evidence for a positive association with walking or cycling. The last decade has seen the construction and testing of walkability indices, at various spatial scales and in different settings, for a wide range of populations. Researchers have tailored components and their quantification, and units of analysis to fit their hypotheses because walkability indices must be designed specifically with the research population and setting in mind [18]. However, three core components—net residential density, street connectivity and land use mix—are salient across populations and form the basis of a majority of indices. Typically, the land uses that are included in the land use mix component to give a measure of the heterogeneity are residential, commercial, institutional and recreational. Christian et al. examined the associations between neighbourhood walkability and walking behaviours in the Perth metropolitan area, Western Australia, using walkability indices that varied in the combination of land uses included [19]. They found that inclusion of only “residential”, “retail”, “offices”, “health, welfare and community” and “entertainment, culture and recreation” gave the strongest association with walking for transport whilst the inclusion of additional land uses such as “public open space” and “sporting infrastructure” gave the strongest association with recreational walking. The associations between all walking—including both transport and recreation—and walkability were slightly stronger with the more comprehensive model.

There is much evidence for positive associations between composite measures of walkability and walking [20–22] but most is from studies of cities in North America and Australia, with less research conducted in European settings. Given difference in urban form, extrapolation of findings to European cities such as London is not appropriate. A review of European studies investigating the relationship between the physical environment and physical activity found results generally concordant with those of non-European studies [23]. However, it noted that measurements of environmental attributes were more often perceived than objective, and that walkability was understudied in European cities. Also, more studies measured total physical activity than walking specifically. A recent investigation of associations between walkability and walking for transport in Stuttgart, Germany, found that individuals living in more walkable neighbourhoods were significantly more likely to spend more time walking for transport per week [24].

### Neighbourhoods

Investigating associations between neighbourhood physical environments and walking also requires consideration of the spatial scale: how the neighbourhood is operationalised to capture exposures in an area that is sensitive to walking. To guide the development of desirable neighbourhoods in industrialising cities, an early spatial definition of neighbourhoods was proposed as a unit that contained a primary school, small parks, small shops, and buildings and streets configured to allow all public facilities to be within safe pedestrian access [25]. However, this definition gives no explicit boundaries, limiting its usefulness to quantitative researchers. The geographical extent of the locations visited is unique to the individual and a product of environmental and individual-level factors [26] such as the proximity of resources and willingness and ability to travel. At present the only means of delineating an individual’s unique neighbourhood is tracking using global positioning systems (GPS) to monitor location of activity, which is impractical for the large study samples needed to detect small but significant effects of neighbourhood physical environments. Compromise is therefore needed through construction or selection of the spatial units to represent a study participant’s neighbourhood. Using GIS software, the neighbourhood may be delineated as a buffer, a boundary placed around an area or a point using a predefined scale as either a straight-line (Euclidean) or a network distance [26]. A Euclidean distance-generated neighbourhood buffer constitutes a circular area with the centre as the individual’s geocoded residential postcode, whilst a network distance-generated buffer is an irregular polygon defined by the street network around the residential location. The distance for buffers can be set such that the area represents a walkable area. The distance that adults walk to reach places from home is approximately one kilometre [9]; surveys indicate that people perceive areas within this distance from home to be part of the neighbourhood [27]. A circular neighbourhood buffer is walkable ‘as the crow flies’, an attribute illustrating a design flaw: a circular buffer, which takes no account of street networks, may contain areas that are invisible or inaccessible to the individual. Therefore, any associations between neighbourhood physical environments and walking may not be found. Whilst network-based buffers may represent a more sensitive neighbourhood representation in this regard, operationalisation of neighbourhoods as either buffer type is often based on the false premise that an individual’s residence is geographically central to their neighbourhood.

Administrative areas constitute the most common operationalisation of neighbourhood in investigations between physical environments and walking because, unlike buffers and GPS traces, they are “ready-made” and often have aggregated environmental measurements available. Also, geolocation of the individual is required only to that area, rather than to a residential postcode. However, the use of administratively defined neighbourhoods can give rise to the ‘modifiable areal units’ problem. This is when different associations are found for the same data when they are aggregated to units of a given shape that differ in size, or of a given size that differ in shape [28, 29]. Whilst not ideal, the use of administrative areas as the spatial units of a walkability index provides independence from the study participants for whom associations with walking are examined: walkability is not limited to the potentially narrow range of participants’ neighbourhoods. Also, a walkability index independent of study participants is potentially applicable to other study populations. Thus spatially contiguous administrative areas are better than participant-centred buffers and GPS traces for neighbourhood operationalisation in the construction of a walkability index.

This study aimed to build a walkability index for London, examining practicalities and the effect of changing the model specification and the units of enumeration on spatial variation in walkability. It also tested the index models through measurement of associations between walkability and walking using data from the Whitehall II Study. It was hypothesised that there would be a gradual radial decay in walkability of London from the centre to the periphery, reflecting reductions in residential dwelling density and street connectivity. A positive association between walkability and time spent walking per week was expected, stronger with the more comprehensive models with regards to land use, and stronger with neighbourhood operationalization at a scale approximating a walkable area.

## Method

### Self-reported walking

The study sample was drawn from Phase 7 of the Whitehall II study conducted in 2003/04. University College London Research Ethics Committee approval was obtained at each study phase. Whitehall II is an ongoing longitudinal study of civil servants to examine the social determinants of health [30]. In 1985, all people between the ages of 35 and 55 years employed in the London offices of the British Civil Service were invited to participate in the study. 73 % agreed to participate, giving a sample size of 10,308 at Phase 1. At 5-yearly intervals, the cohort is invited to a research clinic at which physical examinations are conducted and biological specimens taken. Between these clinic phases, a questionnaire is mailed to participants to collect self-reported data. Geographic residential data including postcodes is collected to maintain contact with participants. These data are useful for examining relationships between health-related behaviours and environmental factors which have an inherent spatial dimension. Phase 7 comprised 6967 individuals (68 % of Phase 1 participants), from whom the 3020 with a valid London postcode and data on physical activity were selected. 38 % of this sample (LWIIP7) was female; the mean age (standard deviation) was 61.0 years (±6.0). The walking volume outcome was derived from the physical activity section of the questionnaire, a modified version of the Minnesota leisure-time physical activity questionnaire [31]. The reliability of the Minnesota questionnaire has been shown to be high [32]. Questionnaire items elicited information on frequency and duration of walking over the past 4 weeks. Walking volume was calculated as the product of duration and frequency of walking. A variable was then constructed, constituting the outcome of being in the top tertile of LWIIP7 for time spent walking per week (TTW) i.e., >6 to 63 h/week.

### Ethics, consent and permissions

Written informed consent from each participant was obtained at each study phase of the Whitehall II study.

### Walkability indices

Walkability indices for London were produced at three spatial scales of contiguous administrative areas: (i) 21,140 output areas, (ii) 633 census area statistics (CAS) wards and (iii) 33 local authorities. At each scale, three walkability models were constructed, as summarised in Table 1, containing the fixed components of residential dwelling density and street connectivity. In addition, each contained a land use mix component which included a different set of land uses. Model 1, the basic model upon which subsequent models were built, comprised the broad land use categories deemed basic personal business destinations potentially reached by foot, namely “Residential”, “Retail”, “Office” and “Health, welfare and community”. Model 2 built on this, adding “Entertainment, culture and recreation” land use. Model 3 extended Model 2 with inclusion of “Free recreational land”, defined as predominantly natural land accessible to all, at no financial cost and potentially suitable for walking for transport or recreation.

**Table 1:** Summary of components included in walkability models

Walkability component		Model 1	Model 2	Model 3
Residential dwelling density		✓	✓	✓
Street connectivity		✓	✓	✓
Land use mix including:	Residential	✓	✓	✓
	Retail	✓	✓	✓
	Office	✓	✓	✓
	Health, welfare & community	✓	✓	✓
	Entertainment, culture and recreation		✓	✓
	Free recreational land			✓

### Data sources

ArcGIS for Desktop Advanced version 10.1 (ArcGIS10.1) [33] was used to store and manage the geographical data with the extensions Productivity Suite, Spatial Analyst and Network Analyst, and the geoprocessing tool, “Line and Junction Connectivity” downloaded under the Esri Toolshare scheme. Attribute data produced by geoprocessing in ArcGIS—tabular or textual data describing the characteristics of geographical features—was transferred from ArcMap to a statistical software package, StataIC (version 12, Stata Corp, College Station, Texas, USA) for statistical processing [34]. Digitised boundary data (aerial representations of geographies to which data can be attributed and visualised) was sourced online from UK Data Service Census Support and used for neighbourhood operationalisation [35]. The spatial data used to compute walkability components was from the UKMap collection, a mapping database product of Landmap [36]. UKMap data is collected by taking 12.5 cm resolution aerial photos that are geometrically corrected using global positioning systems (GPS) and a detailed terrain model from the Cities Revealed LiDAR database. They are then digitised into map features and tested for absolute positional accuracies compared with independent GPS points of 0.7 m random mean squared error accuracy prior to map production. A field survey team walks all areas where there is public access, collecting further qualitative information, which is then compiled into the UKMap database. Finally, a comprehensive specification listing for all captured features is produced, detailing 280 land use codes and eight different feature types.

Data from the UKMap Topo Base data layer, a topographic map depicting the arrangement of artificial and natural features across areas, was used for calculating the land use mix and residential dwelling densities. It was provided in ArcGIS-compatible ESRI format in ninety-one 5 km by 5 km UKMap tiles covering the 1583 km^2^ area of London. These tiles were merged into a single continuous feature class covering London, enabling land use areas to be calculated within the spatial units of enumeration, some of which would span boundaries of UKMap tiles. Ordnance Survey MasterMap (OSMM), a geographical database which digitally represents physical entities such as buildings and roads as topographic features, was used in the calculation of the street connectivity for the walkability index. The particular layers used were the Integrated Transport Network (ITN) layer [37], and the Urban Paths (UP) Theme layer [38], the former provided under licence from Digimap Ordnance Survey Service at EDINA, the national academic data centre based at the University of Edinburgh [39] and the latter provide directly by Ordnance Survey. The ITN layer represents all public and most private driveable roads of Great Britain whilst the UP layer represents the network of transport ways, urban paths, accessible to non-motor vehicle users, including all man-made footpaths, subways, steps, footbridges and cycle paths. Roads and urban paths are topographically represented as links and their junctions as nodes. To calculate street connectivity as a component of the walkability, it was necessary to build a combined ITN and UP layer network dataset. The OSMM data was converted to a format compatible with ArcGIS10.1 using the ESRI ProductivitySuite3 OSMM Data Converter Tool. The OSMM Data Preparation Tool was then used to create a network dataset for the ITN layer and, separately, a network dataset for the UP layer. Subsequently, the Network Analyst extension was used to create a network dataset combining the individual ITN and UP network datasets, henceforth termed the integrated road and path (IRP) network dataset. Residential dwelling counts within each spatial unit of enumeration were obtained from Casweb, a web interface developed and supported by the Census Dissemination Unit [40], to derive the residential dwelling density component of the walkability index.

### Creating the walkability indices

The basis of the computation of core walkability component and final scores were methods described elsewhere [41]. StataIC programmes were written to compute scores for land use mix, street connectivity and residential dwelling density. To derive land use mix scores, the spatial units of enumeration (neighbourhoods) were first associated with co-located polygons depicting land use in the UKMap Topo baselayer by matching rows in the attribute table of the relevant digitised boundaries to those in the UKMap layer based on spatial locations (where a UKMap polygon centroid fell within a boundary). An additional feature layer was produced in which every UKMap polygon was assigned the attributes of the neighbourhood to which it was matched. The area of each UKMap polygon was attributed to the neighbourhood in which its centroid fell—its “host” neighbourhood. The dataset produced by spatial of neighbourhoods and land use polygons, and attribution of land use areas to neighbourhoods, was used to categorise land use mix and calculate its entropy, or “mixedupness”. In order to identify the land uses to be included in the land use mix component, each polygon was assigned a category label based on its specific UKMAP database eight-digit feature classification code. The label assigned indicated whether the polygon represented “Residential”, “Retail”, “Office”, “Health, welfare & community”, “Entertainment, culture & recreation” or “Free recreational land”. Calculations were then made of the total area of a particular land use category, and of the sum of all categories of land use areas, within each neighbourhood. An entropy score for each neighbourhood was calculated according to the following equation (where H = land use mix score, i = the land use, *p*_i_ = the proportion of the area covered by the land use against the sum of the area of the land uses of interest, *n* = the number of land use categories) [19]:

$$ H=-1{\displaystyle \sum_{i=1}^n{p}_i\ast \kern0.5em  \ln \left({p}_i\right)/ \ln (n)} $$

Entropy scores for the neighbourhood covering London were then recoded into deciles with a score of 1 indicative of the lowest entropy and 10 indicating the highest. The indicator of street connectivity was junction density within neighbourhoods. Points identified from the IRP network dataset as representative of street connectivity (those connecting three or more roads or paths) were counted within each neighbourhood and junction density calculated as the number of junctions in a neighbourhood divided by its area. These densities were then recoded into deciles, with neighbourhoods scoring 1 having the lowest junction density and those scoring 10 the highest. Household counts from the 2001 Census, defined as all resident-occupied household spaces within dwellings, were downloaded from Casweb [42], and residential dwelling density for each neighbourhood calculated as the household count divided by the area of land classified as residential dwellings in UKMap within the neighbourhood. Household count data from 2001 was selected based on its temporal proximity to the 2003/04 Whitehall II Study sample data. The raw score for each component of the index was recoded into deciles, with a score of 1 indicative of the lowest entropy or density and a score of 10 indicative of the highest. The overall walkability score was calculated as the sum of the three core walkability component decile scores which was then recoded into deciles and quartiles.

### Statistical analyses and visualisation of walkability

Statistical analysis was performed in Stata to describe the size of the areas, and numbers, of the spatial units defining neighbourhoods and to examine correlations between walkability scores. The Stata dataset was exported to ArcGIS and joined by the geocode of the spatial unit of enumeration to the attribute table of the relevant digitised boundary layer, enabling visualisation of the walkability scores across London. Walkability scores were attributed to each unique LWIIP7 postcode from that of the spatial unit of enumeration in which it fell, and postcode-attached walkability scores were then matched to LWIIP7 participants based on postcode of residence.

Multivariate logistic regression was used to model statistical associations of possible relationships between walkability and walking. In order to model these relationships appropriately, it was necessary to adjust for confounders, factors that are associated with both exposure and outcome but that do not lie on the causal pathway. A literature review conducted for the PhD research project of which this study was part identified various individual level factors—sex, age, economic activity, household car availability—and area-level deprivation as putative confounders of the relationships of walkability with the physical activity outcomes investigated, including time spent walking [43]. Associations were examined by bivariate logistic regression. People aged 66y to 75y were significantly more likely than those aged 50y to <56y to be in the top tertile for time spent walking per week (OR = 1.26; 95 % CI:1.01–1.57; *p* = 0.036). Individuals without access to a car were also significantly more likely to be in this tertile (OR = 1.68; 95 % CI:1.39–2.02; *p* < 0.001) relative to those with car access. Relative to married people, single individuals were significantly more likely to be in the top tertile for time spent walking per week (OR = 1.43; 95 % CI:1.18–1.74; *p* < 0.001). Compared with white individuals, those of non-white ethnicity were significantly less to be in the top tertile (OR = 0.59; 95 % CI:0.46–0.76; *p* < 0.001). There were no significant associations with the other potential confounders, namely sex, economic activity and area deprivation, with being in the top tertile for time spent walking per week. However, the other physical activity outcomes investigated as part of the PhD research from which this study stemmed were significantly associated with these other potential confounders. For consistency, therefore, adjustment was made for all factors identified as potential confounders in modelling the associations between walkability and walking. Bivariate logistic regression models were first specified to determine the presence and strength of significant associations between walkability and walking. Subsequently, adjustment was made for the correlates of physical activity: first, individual level sociodemographic factors and then additionally area deprivation, constructed as England-based quintiles of the 2004 Index of Multiple Deprivation (IMD2004) at lower super output area (LSOA) level [44]. This modelling revealed the extent to which the relationships between walkability exposures and outcomes were independent of other factors. Results were computed as odds ratios alongside their 95 % confidence intervals. The reference category in each model was the lowest walkability quartile score, 1, representing the lowest neighbourhood walkability. Therefore, an odds ratio indicated the odds of being in the top tertile for time spent walking per week for those living in a neighbourhood of higher walkability relative to the odds of this outcome for those in a neighbourhood of the lowest walkability. Tests for trend were performed to evaluate the associations between the outcome and walkability with respect to the trend for a dose effect of the quartile score.

## Results

The smallest administrative units of spatial enumeration for the walkability index were the 24,120 output areas, with a mean (±standard deviation) area of 0.07 km^2^ ± 0.03 and a median area of 0.23 km^2^. Next were 633 CAS wards (mean 2.52 km^2^ ± 2.58, median 1.84 km^2^). The 33 local authorities comprised the largest administrative units of spatial enumeration for the walkability index, with a mean area of 48.30 km^2^ ± 32.80 and a median area of 38.70 km^2^.

Correlations between walkability decile scores of the different models for each spatial unit of enumeration, and between walkability scores of the different spatial units of enumeration for each model, are given in Table 2. For any given administrative geography as the spatial unit of enumeration, correlations between scores of the different models were very high (0.92 to 0.98). Correlations tended to be higher between Models 1 and 2 and between Models 2 and 3 than between Models 1 and 3, for which the difference in the number of land uses included was the greatest. Correlation in walkability scores between local authorities and CAS wards for any given model was moderately high (0.66 to 0.77), but between local authorities and output areas, and between CAS wards and output areas it was lower, a likely manifestation of the greater difference in areal size.

**Table 2:** Correlations between walkability decile scores

		Output areas			CAS wards			Local authorities		
		M1	M2	M3	M1	M2	M3	M1	M2	M3
Output areas	M1	1.00								
	M2	0.97	1.00							
	M3	0.93	0.95	1.00						
CAS wards	M1	0.51			1.00					
	M2		0.50		0.94	1.00				
	M3			0.48	0.92	0.96	1.00			
Local authorities	M1	0.41			0.69			1.00		
	M2		0.41			0.70		0.96	1.00	
	M3			0.39			0.66	0.96	0.98	1.00

**Fig. 1:**
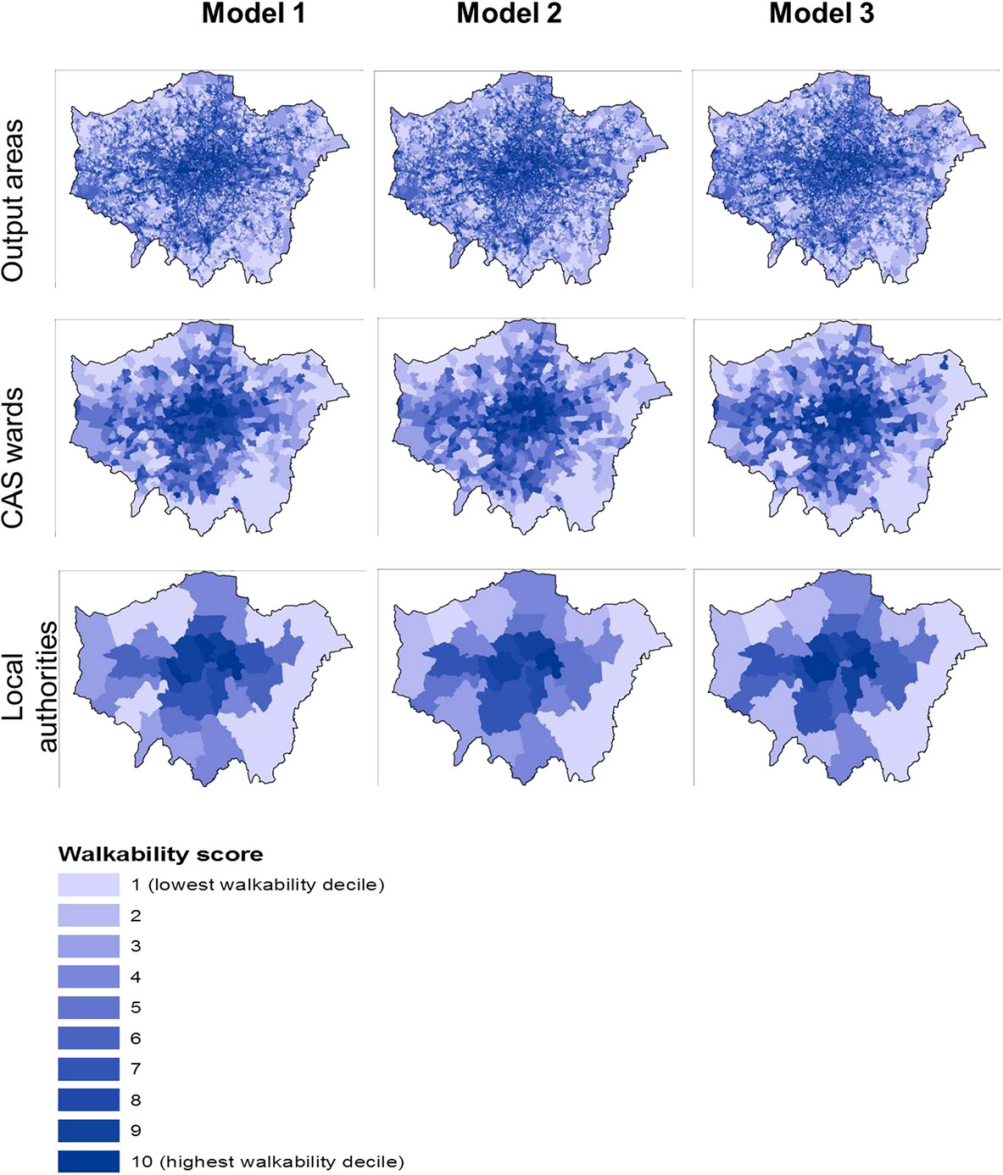
Walkability model maps; Spatial variation in walkability decile scores across London, by spatial units of enumeration and by model

Spatial variation in walkability decile scores across London, by spatial units of enumeration and by model, is presented in the maps of Fig. 1. These illustrate the high inter-model correlation in walkability scores for any given administrative geography as the spatial unit of enumeration that is indicated in Table 2. However, also as indicated in Table 2, Fig. 1 illustrates the lower inter-spatial unit correlation for any given model. Whilst a pattern of radial decay in walkability of London from the centre to the periphery is seen for models for all spatial units, it more uniform and clear for output areas and CAS wards than for local authorities, which constitute relatively large units of geographical data aggregation.

Statistical tests for trend were performed to evaluate overall patterns in the relationships between the walkability exposures and being in the top tertile for time spent walking per week in the LWIIP7 study sample (TTW) before and after adjustment for individual level sociodemographic factors and area level deprivation. The trend test z statistic was positive for all statistically significant models, indicating that participants living in more walkable neighbourhoods had higher odds of TTW than those in less walkable ones. When neighbourhoods were operationalised as local authorities, a significant dose effect of exposure to walkability on TTW was found only for Model 2 and only before adjustment (z-statistic = 2.02; *p* > 0.05). Significant dose effects of exposure to walkability on TTW were also found when neighbourhoods were operationalised as output areas with Models 1 (z-statistic = 2.08; *p* > 0.05) and 3 (z-statistic = 2.22; *p* > 0.05) but, again, only before adjustment. However, when neighbourhoods were operationalised as CAS wards, significant dose effects were found for all three models which remained after adjustment for individual level sociodemographic factors and, additionally, for area level deprivation. Odds ratios for associations between walkability and TTW before and after adjustment for correlates, together with tests for trend, are presented for neighbourhoods operationalised as CAS wards in Table 3. Before adjustment for individual level factors, the highest odds of the TTW outcome were for Model 1 but these were only slightly higher than those for Models 2 and 3. Those living in the most walkable CAS ward-defined neighbourhoods as indicated by a Model 1 quartile score of 4 had higher odds of more time spent walking than those in the least walkable CAS ward-defined neighbourhoods as indicated by a score of 1 (OR = 1.51; 95 % CI:1.19–1.91; *p* = 0.001). After adjustment for individual level sociodemographic factors the odds ratio was 1.39 (95 % CI:1.08–1.79; *p* = 0.010) and, after additional adjustment for area level deprivation, it was 1.42 (95 % CI:1.07–1.89; *p* = 0.015).

**Table 3:** Association between walkability models at CAS ward level and TTW, before and after adjustment

		No adjustment			Adjustment for individual-level factors			Adjustment for individual-level factors & area deprivation		
*N*		2756			2736			2736		
Model	Quartile score	OR	CI	*p*	OR	CI	*p*	OR	CI	*p*
M1	1	1	REF		1	REF		1	REF	
	2	1.04	0.84–1.28	0.729	1.02	0.82–1.27	0.863	1.04	0.82–1.30	0.762
	3	1.29	1.04–1.60	0.018	1.24	0.99–1.55	0.058	1.26	0.99–1.60	0.056
	4	1.51	1.19–1.91	0.001	1.39	1.08–1.79	0.010	1.42	1.07–1.89	0.015
		Test for trend *p* <0.001			Test for trend *p* <0.01			Test for trend *p* <0.01		
M2	1	1	REF		1	REF		1	REF	
	2	1.18	0.96–1.45	0.119	1.15	0.93–1.43	0.192	1.16	0.93–1.45	0.179
	3	1.25	1.01–1.54	0.039	1.19	0.95–1.48	0.135	1.20	0.95–1.52	0.133
	4	1.47	1.14–1.88	0.003	1.33	1.02–1.74	0.033	1.33	0.99–1.80	0.057
		Test for trend *p* <0.01			Test for trend *p* <0.05			Test for trend *p* <0.05		
M3	1	1	REF		1	REF		1	REF	
	2	1.18	0.96–1.44	0.115	1.15	0.94–1.42	0.179	1.18	0.95–1.46	0.142
	3	1.18	0.94–1.48	0.146	1.11	0.87–1.41	0.391	1.13	0.87–1.46	0.351
	4	1.49	1.18–1.88	0.001	1.40	1.09–1.79	0.009	1.41	1.07–1.87	0.015
		Test for trend *p* <0.01			Test for trend *p* <0.05			Test for trend *p* <0.05		

## Discussion

To the authors’ knowledge, this study is the first to construct and test a walkability index for the European city of London based on indices developed in non-European contexts. The significant association between walkability and walking that remained even after adjustment for individual-level sociodemographic factors and for area deprivation represents a novel finding, and one that confirms the validity of the walkability tool constructed in the context of London, UK.

The finding that individuals without access to a car in the household were significantly more likely to be in the top tertile of the study sample for time spent walking per week likely reflects the greater need to walk as a means of transport for those without a car. It is probable that in areas of higher walkability, typified by higher residential density, street connectivity and land use mix, there is less need for access to a car. In line with this theory, individuals without access to a car were found to be significantly more likely to live in more walkable neighbourhoods (data not shown). Of the individual level sociodemographic factors significantly associated with being in the top tertile of the study sample for time spent walking per week, and for which adjustment was made in modelling statistical associations between walkability and walking, household car availability most plausibly accounts for the majority of the attenuation in the relationship. Nevertheless, given that the positive association remained significant after adjustment for sociodemographic variables, it is likely that the propensity to spend more time walking per week is driven at least in part by the walkability of the neighbourhood. Thus, regardless of their access to a car, individuals living in a more walkable neighbourhood would be more likely to spend more time walking. As area deprivation was not associated with being in the top tertile of the study sample for time spent walking per week, it is not surprising that additional adjustment for this factor did not lead to a substantial change in the statistical association between walkability and walking.

This study identified a practical challenge of constructing a walkability index for London in the management of geographical data within separate GIS and statistical software packages. Due to limitations in the statistical capabilities of the GIS software, there was a need to transfer and reformat data, a process prone to error. Another difficulty highlighted was indexing walkability within the very small administrative units of output areas: it was not possible to index walkability in accordance with the method specified as a result of the particularly small size of these administrative units relative to areas of land uses included in the land use mix component. Land use mix entropies could not be quantiled into deciles for output areas because more than 10 % had an entropy of zero, the manifestation of a spatial unit containing none of the land uses specified in a particular land use model, or being covered entirely by one land use.

The a priori expectation that there would be radial decay in walkability of London from the centre to the periphery held true. This reflected reductions in residential density and junction density, two of the core walkability components. Very high inter-model correlations in walkability scores found at any given administrative geography as the spatial unit of enumeration suggested that neighbourhoods with a “good” mix of the basic land uses of Model 1 – defined as “Residential” “Retail” “Office” “Health, welfare & community” – also had well-mixed land use when uses were extended to encompass the “Entertainment, culture and recreation” of Model 2 and, the “Free recreational land” of Model 3. The implicit co-location of land uses fits Wegener and Franz’s “Land-use transport feedback cycle”, in which land development sparks further development [45].

The finding that for a given model, correlations in walkability scores between CAS wards and the local authorities in which they fell were higher than those between output areas and local authorities in which they fell is consistent with Tobler’s “first law of geography”, a phenomenon where “everything is related to everything else, but near things are more related than distant things” [46]. The difference in the median areal size between output areas and local authorities was greater and thus it was more likely that a local authority would contain walkability-related attributes from which its score was derived that differed from those of the output areas it contained, leading to a divergence in walkability scores.

As hypothesised, living in a more walkable neighbourhood was associated with being in the top tertile for time spent walking per week in LWIIP7 (TTW) because walkability is supportive of walking. Given the high inter-model correlation in walkability scores, it is not surprising that the significant positive associations found between walkability and TTW, after adjustment for individual-level sociodemographic factors and for area deprivation, did not differ markedly for different models. Also as expected is the finding that the only significant associations remained between walkability and TTW after adjustment for correlates when neighbourhoods were operationalised as CAS wards. The median area of these neighbourhood operalisations, at 1.84 km^2^, approximates the size of a 1 km walkable neighbourhood, assuming a circular buffer delineation (3.14 km^2^). In contrast, the scale of the smaller output areas and the larger local authorities differed from such a delineation by an order of magnitude. Notwithstanding the modifiable areal unit problem inevitably presented in the use of administrative areas as neighbourhoods for measurement of walkability, this study demonstrates that CAS wards may constitute suitable spatial units of enumeration for walkability. The reduction in the strength of significant associations between walkability and walking after adjustment for individual-level sociodemographic factors and for area deprivation is consistent with strong evidence for the role of such factors in physical activity behaviours [47].

### Limitations

The core components of the walkability index and the land uses included in the land use mix part were not weighted to reflect their hypothesized relative importance, a procedure that is advocated by others [48]. However, in the novel UK city context in which this study was set, there was scant evidence on which to base such weightings. Whilst significant relationships between walkability and walking were found, causality could not be inferred due to the cross-sectional study design. Even if this study had had a longitudinal design and showed that moving to a more walkable area resulted in greater walking, this could be due to self-selection, with the reason for the move or the choice of location when moving being influenced by a desire to walk more [49]. Also, participants did not report the location of their self-reported walking so it may have been independent of the neighbourhood exposure. Measures of physical activity in the 2004/5 wave of the Whitehall II study from which the sample for this research was drawn were self-report and, as such, likely to incur substantial imprecision. In the 2012/2013 wave, physical activity in the Whitehall II cohort was additionally measured objectively by accelerometry. A moderate correlation between self-reported and objectively measured physical activity was found [50]. Correlations were higher for people of higher socioeconomic status—as defined by occupational position and education—and for more energetic physical activities. Whilst measurement of physical activity by accelerometry is generally more accurate for assessing duration and intensity of activity, these findings suggest the use of self-reported physical activity data in the present study was adequate for testing associations with walkability. It should also be noted that health-based recommendations for activity levels are based on self-reported physical activity.

### Strengths

The Whitehall II Study had a very high response rate, enabling the use of a large study sample. This limited the influence of outliers as extreme observations and allowed detection of statistically significant associations that may not have been detectable with smaller samples. The high quality and large quantity of data collected in the Whitehall II Study also allowed adjustment for a multitude of sociodemographic factors for which there is evidence for association with walking. Identification of participants to postcode-level enabled examination of the effects on associations of neighbourhood operationalisation at a wide range of scales, a privilege enjoyed by few researchers using large study samples in this field of study. The administrative boundary and Census data used in the calculation of walkability was of high quality and freely available, reducing the financial cost of producing the index. Also, the use of high quality of the road and path network data, sourced from a well-established organisation which is one of the world’s largest producers of maps, provided confidence that the measures of walkability were accurate.

## Conclusions

In the context of the most populous city in Europe, the significant association between walkability and walking, even after adjustment for individual-level sociodemographic factors and for area deprivation, highlights the potential importance of the physical environment of the neighbourhood in eliciting physical activity in individuals and thereby promoting public health at a population level. The most basic walkability index model constructed here may offer urban planners and public health professionals a simple tool in building and maintaining healthy neighbourhoods.

In future work, the walkability index could be used to assess relationships between walkability and walking in regionally or nationally representative samples, and for different age groups. Previous work revealed lack of associations between walkability and overall physical activity in the study cohort, suggesting a neighbourhood supportive of walking is not necessarily conducive to other physical activities [43]. Therefore, investigation of associations of walkability with specific types of physical activity within particular domains using a regionally or nationally representative sample is warranted.

## Availability of data and materials

Permission to use data collected in Phase 7 of the Whitehall II study can be sought from University College London (http://www.ucl.ac.uk/whitehallII/data-sharing). Sources of the data used in construction of the walkability index are provided under Data Sources in the Methods section.

## Abbreviations

CAS ward: census area statistics ward
CI: confidence interval
DALYs: disability-adjusted life-years
GIS: Geographic Information System
GPS: global positioning system
IMD: Index of Multiple Deprivation
IRP: integrated road and path
ITN: Integrated Transport Network
LSOA: lower super output area
LUM: land use mix
LWIIP7: London-dwelling Whitehall II Study Phase 7
M1: Walkability model 1
M2: Walkability model 2
M3: Walkability model 3
OR: odds ratio
OSMM: Ordnance Survey MasterMap
SD: standard deviation
TTW: being in the top tertile of LWIIP7 for time spent walking per week
UP: Urban path
